# Accuracy of ChatGPT 3.5, 4.0, 4o and Gemini in diagnosing oral potentially malignant lesions based on clinical case reports and image recognition

**DOI:** 10.4317/medoral.26824

**Published:** 2025-01-26

**Authors:** Pragya Pradhan

**Affiliations:** 1 MDS, Public Health Dentistry, Department of Dentistry, District Hospital, Madhya Pradesh, India

## Abstract

**Background:**

The accurate and timely diagnosis of oral potentially malignant lesions (OPMLs) is crucial for effective management and prevention of oral cancer. Recent advancements in artificial intelligence technologies indicates its potential to assist in clinical decision-making. Hence, this study was carried out with the aim to evaluate and compare the diagnostic accuracy of ChatGPT 3.5, 4.0, 4o and Gemini in identifying OPMLs.

**Material and Methods:**

The analysis was carried out using 42 case reports from PubMed, Scopus and Google Scholar and images from two datasets, corresponding to different OPMLs. The reports were inputted separately for text description-based diagnosis in GPT 3.5, 4.0, 4o and Gemini, and for image recognition-based diagnosis in GPT 4o and Gemini. Two subject-matter experts independently reviewed the reports and offered their evaluations.

**Results:**

For text-based diagnosis, among LLMs, GPT 4o got the maximum number of correct responses (27/42), followed by GPT 4.0 (20/42), GPT 3.5 (18/42) and Gemini (15/42). In identifying OPMLs based on image, GPT 4o demonstrated better performance than Gemini. There was fair to moderate agreement found between Large Language Models (LLMs) and subject experts. None of the LLMs matched the accuracy of the subject experts in identifying the correct number of lesions.

**Conclusions:**

The results point towards cautious optimism with respect to commonly used LLMs in diagnosing OPMLs. While their potential in diagnostic applications is undeniable, their integration should be approached judiciously.

** Key words:**Oral premalignant lesions, oral diagnosis, natural language processing, mouth neoplasm, computer-assisted diagnosis.

## Introduction

When the renowned mathematician and computer scientist Alan Turing posed the question, "Can machines think?", it marked the beginning of Artificial Intelligence (AI) as a field dedicated to understanding human-like intelligence and attempting to replicate it. In November 2022, the field of AI achieved a milestone with the launch of Chat Generative Pre-Trained Transformer (ChatGPT) by OpenAI. OpenAI is an American AI research organization primarily focused on building generative models using deep learning technology. Shortly after, Google launched Bard in March 2023, which was later transitioned to Gemini in December 2023. Both, ChatGPT and Gemini, are essentially chatbot technology with the ability to process natural human language and generate coherent and contextually suiTable response. The models are pre-trained on large amount of textual data which is then used for targeted applications such as text classification and question-answering. In the pre-training phase, models are trained on language modelling task, which involves predicting the next word in a text sequence based on the previous words in the sequence (1).

The efforts to integrate Natural Language Processing (NLP) with healthcare is not new and has seen an increase in research over the past 20 years (2). NLP techniques have been shown to manage medical information overload, while also assisting in medical decision making by examining the similarities and differences within vast amounts of text data. Yet, the launch of ChatGPT, a result of NLP advancements, managed to catch the eyes of the world due to its browser based, user-friendly interface, which facilitates easy interaction with the language-based learning model, regardless of technical expertise (3). Its multifaceted role in healthcare practise and research includes, but is not limited to, gathering, analysing and interpretating data, writing and editing scientific literature, as an aid in medical teaching, patient education, streamlining clinical workflow, and assisting in diagnosis and treatment planning (4). The advantages associated with the tasks includes accessibility, efficiency, improved communication, language assistance and reduced cost (5). However, its handling have concerns in the scientific community regarding limited knowledge, risk of bias, plagiarism, lack of originality and ethical issues (5). In addition, ChatGPT lacks critical thinking and reasoning, hence, often produces incorrect information with factual inconsistencies, a phenomenon known as hallucination. With the possible advantages and disadvantages, the various uses of ChatGPT are currently under scientific scrutiny and lacks a general consensus (6).

Artificial intelligence encompasses a variety of technologies, including machine learning. The most advanced form of machine learning is deep learning, which utilizes multiple layers of variables to predict outcomes. Clinical decisions are often guided by the clinician’s expertise; however, the constant expansion of study material and emergence of complex cases calls for improved diagnostics. In healthcare, deep learning is commonly applied to enhance diagnostic accuracy and support precision medicine. Large Language Models (LLMs) like ChatGPT and Gemini, which are based on deep learning, has demonstrated its utility as a supplementary tool for clinical decision-making in both straightforward cases and complex clinical vignettes (7). A major concern worldwide is the increase in incidence of oral cancer. Multiple factors including prognosis, morbidity and mortality associated with oral cancer hinges upon its early diagnosis at the pre-malignancy stage of the lesion (8). These lesions carry a malignancy risk, with transformation rates varying between 13% and 90% (9). A significant challenge associated with prompt detection is diagnostically challenging nature of Oral Potentially malignant Lesions (OPMLs) and our limited understanding to distinguish high-risk OPMLs from low-risk lesions (10). Moreover, a definitive diagnosis of OPMLs requires biopsy, which some practitioners might be hesitant to undertake due to methodological obscurity, lack of training and limited knowledge of OPMLs (11,12). Various OPMLs also present with overlapping clinical features (9). Additionally, the prospect of a biopsy may provoke anxiety and discomfort among patients (13). Therefore, it is imperative to explore the potential of ChatGPT as an adjunct in clinical decision-making. Previous studies have reported inconsistencies in ChatGPT’s response with general guidelines and consensus regarding knowledge, diagnosis and risk factors associated with OPMLs (14). Nonetheless, ChatGPT has shown to be an effective resource for providing patients with information on the early identification of oral cancer (15). The author observed a gap in literature regarding assessing the accuracy of LLMs such as ChatGPT and Gemini in diagnosing OPMLs based on clinical case reports. Hence, this study was performed with the aim to assess the accuracy of ChatGPT 3.5, 4.0, 4 Omni (4o) and Gemini in diagnosing OPMLs based on Clinical Case Reports. In addition, ChatGPT 4o and Gemini is also evaluated on its diagnostic abilities based on image recognition.

## Material and Methods

- Search Strategy

A list of Oral Potentially Malignant Lesions was prepared following the classification by Warnakulasuriya S *et al*. (16). A comprehensive search was carried out in PubMed, Scopus and Google Scholar for published reports of cases corresponding to the classification. The reports were screened for their relevance to the classification and included only if they met the following conditions: they contained a detailed patient history, symptoms, examination findings, and a confirmed diagnosis through standard procedures; they provided a clinical description of the lesion that matched the images available in the database; they were primarily open access to ensure fair use in research, though subscription-based articles were included when highly relevant; and they were written in English. Additionally, reports were selected to represent a diverse range of demographics and geographic locations. A total of 42 case reports were selected for final analysis, which included 34 OPMLs and 8 Oral Squamous Cell Carcinoma (OSCC) case reports to allow for direct comparison between malignant and premalignant stages. Of the 34 OPMLs reports, there were 4 cases each of Oral Leukoplakia, case reports of variants of Oral Lichenoid Lesions (OLL) and Oral Lupus Erythematosus (OLE) among others. The four variants of Oral Lichen Planus (OLP) were included based on their most common occurrence.

- Image Recognition Case Reports

In addition to testing the abilities of GPT 3.5, 4.0, 4o and Gemini based on text recognition, the recently launched GPT 4o and Gemini were also assessed for their abilities to diagnose cases based on a combination of text and image. Publicly available collection of OPMLs photos (https://opmdcare.com/atlas-photos/) and OSCC photos (https://oralcancerfoundation.org/dental/oral-cancer-images/) were accessed in June 2024. The images were selected corresponding to the case reports. Additionally, images for following conditions, namely, Papular OLP, Oral Lichenoid Drug Reaction, Discoid Lupus Erythematosus, Systemic Lupus Erythematosus, Dyskeratosis Congenita, OSCC of Gingiva, and Verrucous Carcinoma were sourced from open access case reports.

- Reports Preparation

The case reports were anatomized and only the history section was included for input in different LLMs. The case description included information on the patient’s demographics, chief complaint, history of presenting illness, any medical/dental/drug history, personal history such as tobacco usage, description of clinical lesion, any associated symptoms and histopathological findings. The case reports used for image analysis were parsed to match with the lesion characteristics and its location in the image. The reports were entered into ChatGPT and Gemini text box with the additional prompt: “Based on the above description, what is the most likely diagnosis?” For image recognition cases, the case reports were followed by the prompt “Based on the above image and case description, what is the most likely diagnosis?”

In order to overcome the memory retention bias and also the possible reinforcement learning from human feedback (RLHF) capabilities demonstrated by LLMs, each question was processed in a new chat session. During the course of this study, OpenAI launched memory feature in ChatGPT, which allowed GPT to remember information. However, this feature was turned off to prevent any additional learning by GPT. The case reports and images were also submitted to two Subject Experts (SEs) for their evaluation. The accuracy of the provisional diagnosis was calculated by categorizing the responses into ‘correct’, ‘partially correct', and ‘incorrect. The study did not require ethical approval as per the local Institutional Review Board (IRB), but it still adhered to ethical considerations relevant to the handling of data and use of secondary information.

- Statistical Analysis

The data was entered into Microsoft Excel and analysed using SPSS Version 25 (IBM Corp., Armonk, New York, United States). Categorical data was analysed by Friedman’s Test to detect differences in rankings across multiple groups. It was followed with pairwise comparison by Dunn’s Post-hoc adjusted for Bonferroni correction for multiple tests. The data was also assessed for interrater agreement measured by Cohen’s kappa (κ).

## Results

**Figure 1:**
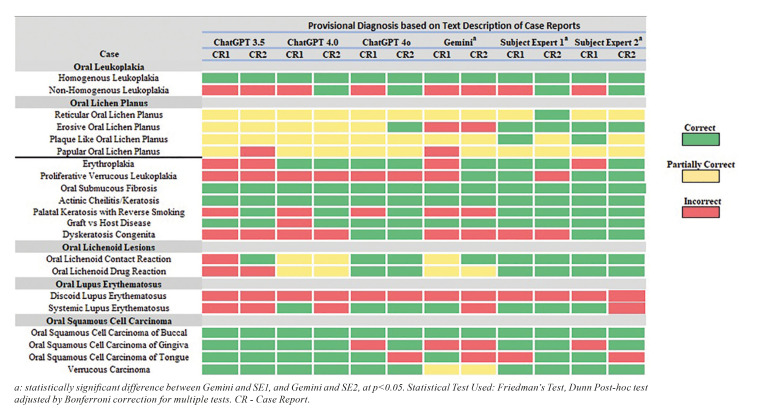
Provisional diagnosis by ChatGPT 3.5, 4.0, 4o, Gemini, Subject Expert 1 and 2 based on text description of case reports.

Fig. 1 shows the provisional diagnoses from four LLMs and two SEs, using a heatmap based on text descriptions from case reports.

**Figure 2:**
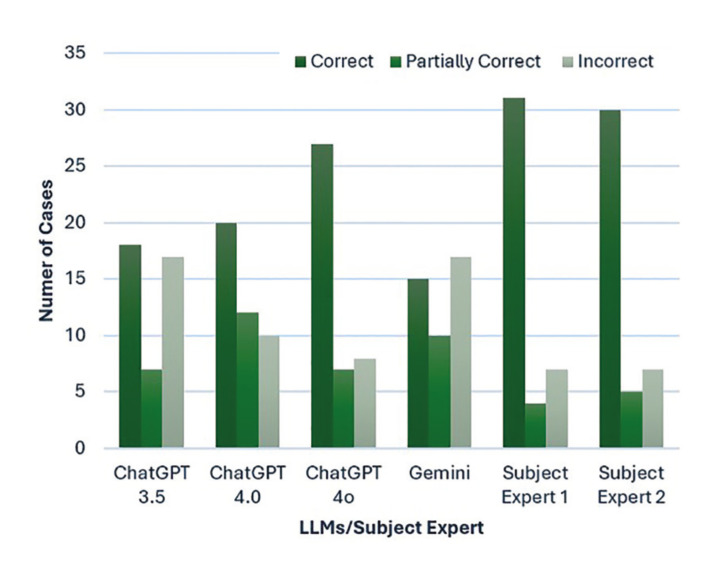
Distribution of responses by ChatGPT 3.5, 4.0, 4o, Gemini, Subject Expert 1 and 2 to text description of case reports.

The frequency distribution of responses indicates that GPT 4o aligns most closely with the subject experts in accurately identifying OPMLs, followed by GPT 4.0 and GPT 3.5. Google’s Gemini recorded the fewest correct diagnoses. It also recorded the highest number of incorrect diagnoses along with GPT 3.5. Furthermore, Gemini's responses demonstrated a statistically significant difference from those of subject experts 1 & 2 (*p*<0.05). There was also some overlap between GPT 3.5 and 4o in terms of partially correct responses. Both subject experts had a similar number of incorrect responses. (Fig. 2).

**Figure 3:**
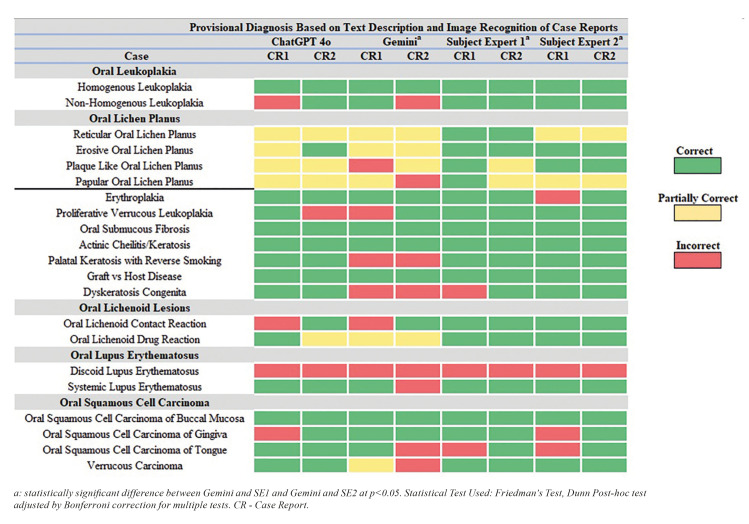
Provisional diagnosis by ChatGPT 4o, Gemini, Subject Expert 1 and 2 based on text description and image recognition.

**Figure 4:**
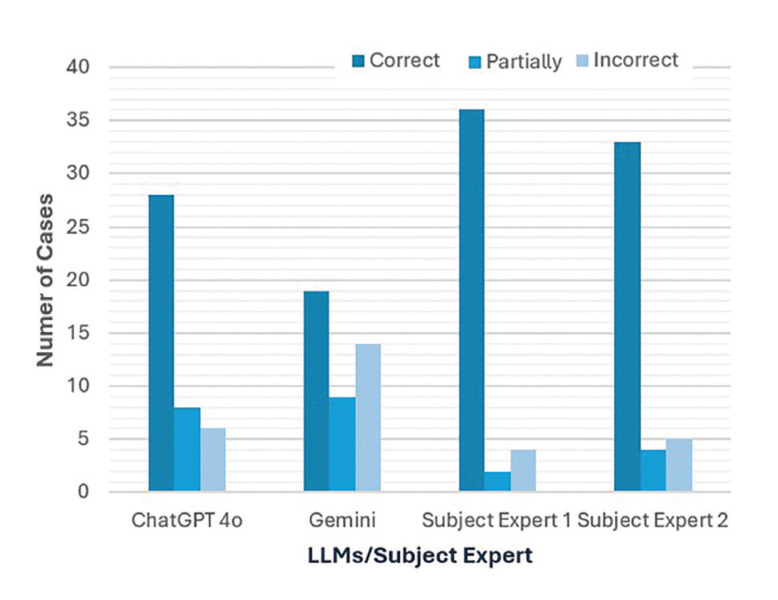
Distribution of responses by ChatGPT 4o, Gemini, Subject Expert 1 and 2 to text description and image recognition of case reports.

Fig. 3 shows scenarios involving image recognition in which GPT 4o accurately identified 28 out of 42 cases (66.6%), while Gemini correctly identified 19 out of 42 cases (45.2%). GPT 4o made incorrect recognitions in 6 out of 42 cases (14.2%), compared to Gemini, which incorrectly identified 14 out of 42 cases (33.3%). (Fig. 4) Multiple group response comparison revealed statistically significant differences in response between Gemini and Subject Expert 1 and 2 (*p*<0.05).

The data was further analysed to evaluate the agreement between the LLMs and human experts by calculating Cohen’s kappa (κ). The results indicated a substantial agreement between GPT 4o and SE2 (κ = 0.659) and moderate agreement between GPT 4o and SE1 (κ = 0.543) and GPT 4.0 and SE1 (κ = 0.468). The results also showed fair agreement between GPT 3.5 and SE1 (κ = 0.365) and SE2 (κ = 0.333) and between GPT 4.0 and SE2 (κ = 0.350). There was a fair level of agreement between Gemini and SE1 (κ = 0.326) and a moderate level of agreement between Gemini and SE2 (κ = 0.413). For image recognition, there was moderate agreement between GPT 4o and SE2 (κ = 0.514), while there was fair agreement between GPT 4o and SE1 (κ = 0.396), Gemini and SE 1 (κ = 0.265) and SE2 (κ = 0.245). All the kappa values were statistically significant at *p*<0.05. (Table 1).

## Discussion

A widely prevalent form of contemporary artificial intelligence is deep learning, which uses multi-layered deep neural networks to simulate the complex decision-making capabilities of the human brain. The core architecture of Conversational AI is based on the Transformer model which facilitated the development of advanced Large Language Models (LLMs) such as OpenAI’s ChatGPT 3.5, 4.0 and 4o as well as Google’s Gemini. Since their launch, AI chatbots have advanced substantially, evolving from simple scripts to complex conversational systems. Recent studies have shown that ChatGPT exhibited significant accuracy and remarkable competency in the Medical Licensing Exams of countries such as USA, Peru, Saudi Arabia and Germany (17). In addition to medical knowledge enquiries, LLMs have shown high diagnostic accuracy in predicting medical scenarios. However, it is not without reliability concerns. Even though arriving at a diagnosis is a complex process and requires analysing vast amount of patient’s history, medical history and laboratory test results, ChatGPT has demonstrated high accuracy in diagnosing both basic and complex cases requiring specialized knowledge (7).

In the present study, various models of LLMs were compared in diagnosing OPMLs based on clinical case reports and image recognition. Using only the text description, Subject Expert 1 and Subject Expert 2 achieved 73.8% (31/42) and 71.4% (30/42) correct responses, respectively, while ChatGPT 4o correctly identified 64.2% (27/42) of the lesions. This was followed by ChatGPT 4.0 which got 47.6% (20/42) responses correct and ChatGPT 3.5 which correctly identified 42.8% (18/42) of the cases. Among LLMs, Gemini got 35.7% (15/42) correct responses. Furthermore, improvement in accuracy of both GPT 4o and Gemini was observed when images were also inputted along with case description. GPT 4o correctly identified 66.6% (28/42) of the cases whereas Gemini had 45.2% (19/42) correct responses. Improvement in recognizing cases of Proliferative Verrucous Leukoplakia (PVL), Palatal Keratosis with Reverse Smoking and OSCC of tongue was found with GPT 4o. While Gemini showed some improvements in correctly identifying Non-Homogenous Leukoplakia, Erythroplakia and OSCC of Gingiva when provided with an image. The inclusion of images provided critical visual information that enhanced accuracy. In text-only descriptions, GPT 4o’s diagnoses, although incorrect, were closer to the differential diagnosis compared to other models (18).

The categorization of responses was not dichotomous; instead, it included a third category of 'partially correct.' This approach allowed for a more nuanced evaluation of the performance of LLMs by including those diagnoses that captured essential elements of the correct response but may have missed certain details. In the present study, responses were categorized as partially correct if the lesions were not accurately identified along with their subtypes or if they were identified as OSCC, considering Verrucous Carcinoma is a low-grade variant of OSCC (19). Interestingly, based on text description, both GPT 3.5 and GPT 4o recorded partially correct responses for 16.6% (7/42) of cases, whereas GPT 4.0 had 28.5% (12/42) and Gemini had 23.8% (10/42) of responses classified as partially correct. These responses predominantly involved cases of Oral Lichen Planus and Oral Lichenoid Lesion, where the models accurately identified the lesion but failed to specify its subtypes. This could be because the variants are often intermingled with respect to their characteristic features and presenting symptoms (20). Compared to the LLM models, the SEs accurately identified the lesions and their subtypes, only partially misidentifying variants of OLP from text descriptions. However, when presented with images, the SEs corrected their diagnoses, while both GPT 4o and Gemini either partially identified the lesion or got the diagnosis incorrect.

LLMs like GPT and its variants are designed using intricate architectures that process vast amounts of data through Non-Supervised Pre-Training. The high number of parameters that they are trained on (GPT 3 with 175 billion, GPT 4 with significantly more, Gemini; Not known) contributes to the model's ability to understand the pattern better (21). While GPT 3.5 operates on a fixed dataset with a training cut-off in 2021, both ChatGPT 4.0 and 4o include data up to December 2023. In contrast, Gemini has the capability to access real-time internet data. In the present study, 76.1% (32/42) case reports were pre 2021 and 23.8% (10/42) were published after 2021. Among these, 38 case reports were accessed through open access sources, and four required subscription access. It is noteworthy that Gemini and GPT 3.5 had the highest number of incorrect diagnoses, followed by GPT-4.0, and GPT-4o. The progression from GPT-3.5 to GPT-4o includes improvements in the underlying algorithms, leading to a better understanding of medical information, which is evident in the results. In contrast, Gemini’s access to real-time internet data can introduce a high amount of unverified or conflicting information, thereby increasing the likelihood of incorrect diagnoses. Similar findings were reported in a study by Shukla R. *et al*. (22) on neuro-ophthalmology cases. It is also important to point that none of the LLMs correctly identified OPMLs part of systemic dysfunction such as Discoid Lupus Erythematosus. Additionally, although all LLMs correctly diagnosed Homogeneous Leukoplakia, only GPT 4.0 and GPT 4o accurately identified cases of Non-Homogeneous Leukoplakia.

The expansion of AI into healthcare has garnered a range of responses from medical professionals. Concerns include inadequate knowledge of AI and fear of replacement and displacement. There is also reluctance to incorporate AI into clinical practice, coupled with skepticism about the system's quality and effectiveness in diagnostic processes (23). However, attitudes are not uniformly negative. Many professionals have expressed positive views toward AI's potential, particularly in diagnostic specialties. Pathologists, for example, advocate for AI's role in crafting personalized treatment plans (24). In oral diagnostics, various models of AI have demonstrated efficiency in identifying OPMLs and OSCCs. Furthermore, LLMs such as ChatGPT are being considered to play an important role in risk prediction model of OPMD/OSCC. A study done by Islam A *et al*. (25) reported high sensitivity of GPT 3.5 response to oral pathology queries, and presented substantial agreement with the experts. In our study, however, we predominantly found fair to moderate agreement between the SEs and LLMs on their responses. Diagnosing OPMLs and OSCCs can be challenging requiring clinicians to undergo extensive training over many years to accurately identify and manage these conditions. Therefore, despite the sophistication of LLMs, it may not demonstrate the originality, creativity, and critical thinking necessary for diagnosing these conditions (6). This perspective is further reinforced by patients consistently expressing a strong preference for retaining human oversight in medical decisions (26).

AI-assisted diagnostics face significant challenges, which includes a lack of standardization, ethical dilemmas, biases, legal concerns, and the scarcity of publicly available databases for training. Other issues, such as interpretability and adaptability, also play crucial roles. Additionally, LLMs may have cost, readability, and accessibility related constraints (27). Many of these limitations overlap. AI models are developed using various methodologies and datasets, resulting in variance in quality and comprehensiveness based on the source. It can sometimes intensify human biases leading to underdiagnosis for population groups, such as females, black patients, and patients of lower socioeconomic status (28). A limited number of publicly accessible database for image analysis of OPMDs and OSCCs presents another challenge. In a study by N. Sengupta *et al*. (29) the authors found only one image database for oral cancer. In the present study, although all the images were sourced either from publicly accessible databases or from open access articles, there were only minor improvements in diagnosis through image recognition by GPT 4o and Gemini. In fact, in one of the misdiagnosed cases, Gemini provided the reference link for the source of the image. This outcome raises questions about the adequacy of the training data used for these models, especially when it comes to image recognition. AI-assisted clinical decision making also brings to light several ethical considerations, including the issues of accountability, transparency on AI functioning, securing informed consent for data use, and protecting patient’s privacy (30).

This study is not without its limitations. One of them is the limited sample size, consisting of 42 case reports, with 2 cases corresponding to each OPML. This limited selection was due to the inadequate number of publicly available image database for OPMLs to correspond to the case reports. Additionally, the study compared only the popular LLMs for their diagnostic abilities. LLMs such as Claude and Perplexity, may offer diverse performance based on the variation in their training data. Including a wider variety of LLMs with larger sample size in future research could provide a more comprehensive understanding of their diagnostic potential.

## Conclusions

The results of the present study points towards cautious optimism regarding AI-assisted diagnosis of OPMLs. The analysis found that among LLMs, GPT 4o identified the highest number of lesions correctly followed by GPT 4.0, GPT 3.5 and Gemini. In diagnosing OPMLs through image recognition, GPT 4o outperformed Gemini with the greatest number of correct responses. However, it is worth mentioning that Gemini provides the option of image analysis in its free version, whereas it is a paid service included in ChatGPT 4o. This can have significant impact in terms of affordability and accessibility. Gemini, GPT 4o and GPT 4.0 also provided a range of differential diagnosis along with their responses. Despite these advances, all LLMs fell short of matching the diagnostic precision of subject experts. Nevertheless, including partially correct responses as a factor enhances the perceived diagnostic capabilities of LLMs. When taking assistance of conversational AI-bots, clinicians should be aware of its limitations in correctly identifying the subtypes of various OPMLs and should anticipate receiving general responses rather than specific insights. Its application can be particularly useful in remote settings with limited manpower and resources. ChatGPT and Gemini can be used to analyse patient’s history, risk factors, and clinical presentation of OPMLs and aid in clinical decision-making process. The findings of the present study further reinforce the need of human supervision for AI-assisted diagnosis. Clinical decision-making involves numerous factors, at the core of which is human engagement, which cannot be replaced by Artificial Intelligence.

## Figures and Tables

**Table 1:** Interrater agreement between the Subject Experts and ChatGPT 3.5, 4.0, 4o and Gemini.

AI Model - Subject Expert	Cohen's Kappa	
	Text Description	Text + Image Identification
ChatGPT 3.5 - SE1	0.365*	NA
ChatGPT 3.5 - SE2	0.333*	NA
ChatGPT 4.0- SE1	0.468*	NA
ChatGPT 4.0 - SE2	0.350*	NA
ChatGPT 4o - SE1	0.502*	0.396*
ChatGPT 4o - SE2	0.659*	0.514*
Gemini - SE1	0.326*	0.265*
Gemini - SE2	0.413*	0.245*

LLM - Large Language Model; SE - Subject Expert; NA - Not Applicable *Statistically significant at p<0.05.

## Data Availability

Derived data supporting the findings of this study are available from the Correspondence on request.
